# Identification of GPAT acyltransferases in cork oak

**DOI:** 10.1186/1753-6561-5-S7-P69

**Published:** 2011-09-13

**Authors:** Liliana Marum, Andreia Miguel, Pinto C Ricardo, Célia Miguel

**Affiliations:** 1 Instituto de Tecnologia Química e Biológica-Univ. Nova de Lisboa; Instituto de Biologia Experimental e Tecnológica (ITQB-UNL;IBET), Av. República, EAN, 2780-157 Oeiras, Portugal

## Background

Acyltransferases are enzymes with an important role in the synthesis of both cutin and suberin which are part of the lipophilic barriers, such as epidermis and periderm that protect terrestrial plants against water loss and other external aggressions. During secondary growth in woody plants such as cork oak (*Quercus suber* L.), the epidermis is replaced by a suberized periderm that includes the phellem (cork), phellogen (cork cambium) and phelloderm tissues. In *Q. suber* the successive formation of phellem following removal at periodic intervals (every 9 years) allows for exploitation of cork oak on a sustainable basis. The main component of cork (45-50%) is suberin, a complex polymer comprising both aliphatic and aromatic domains and associated waxes [1,2]. Despite the physiological importance of suberin, its biosynthetic pathway as well as its deposition remains largely unknown. Since cork oak is a unique species among terrestrial plants due to its remarkable capacity for cork production, it is expected that suberin biosynthesis and deposition are tightly controlled mechanisms.

As a first step to start unraveling these control mechanisms we intend to identify and characterize genes coding for the acyltransferases of the GPAT (glycerol-3-phosphate acyltransferase) family, involved in suberin and cutin synthesis in cork oak. Two ESTs highly similar to *GPAT5* (EE 743864 and EE 743865) and one EST (EE743668) highly similar to *GPAT4* shown to be strongly up-regulated in the suberin-rich phellem of cork oak tree (*Q. suber*) were first identified by Soler et al. [3].

## Material and methods

In this work, phellem tissues from small branches with increasing age (1 to 7 years old) were harvested from cork oak and holm oak (a related but cork non-producing species)at the Instituto Superior de Agronomia (Portugal). Tissues collected during different growth periods were also used for analysis: samples collected during a period of high phellogen activity (April – June, 2009 and 2010) and samples collected during the inactive growth period (January, 2010). Total RNA was successfully extracted from these tissues using a protocol described by Reid et al. [4], with minor modifications. cDNA was synthesized using standard procedures and 5’- and 3’-RACE are being performed in order to determine the full-length of putative *GPAT* coding sequences from *Q. suber* transcriptome. The expression level of *GPAT4* and *GPAT5* genes was assessed by quantitative RT_PCR in two different seasonal stages (April and June) in periderm cells from 3 year old branches of *Q. suber*. The Cp values were converted into relative quantities, using the formula, Q=E^∆Cp^, where E (the efficiency of the gene amplification for each primer pair) was calculated using the Real-time PCR Miner algorithm.

## Results and conclusions

A cDNA fragment with 1265bp of the putative *Qs_GPAT5* was obtained. The predicted amino acid sequence displays the glycerol-3-phosphate acyltransferase (PLN02499) conserved domain. Based on a database search we have identified putative orthologs of *AtGPAT5* in *P. trichocarpa* (Pt_8s_AT, accession number 002312108; Pt_10s_AT, accession number 2315213) and *R. communis* (Rc_ERGPAT, accession number 2531580) genome, which show high similarity to the putative *Q. suber GPAT5* gene. The *Rc_ERGPAT* is most similar to the *Qs_GPAT5* with an identity of 83% at the amino acid level.

The expression profiles of *GPAT4* and *GPAT5* were successfully analysed during phellem differentiation in periderm collected from cork oak tree. The relative expression level of *GPAT4* gene was similar in April and June. However, the relative expression level of *GPAT5* gene was higher in June, which corresponds to a period of higher phellogen activity, when compared to April.

Further information on the expression of these genes in several tissues of cork oak under different developmental stages and stress conditions will also be gathered as a result of the recent effort of the Portuguese research community involved in the transcriptome sequencing of cork oak. With this work we expect to contribute to elucidate basic aspects of the molecular networks involved in cork formation.
